# Long Distance Movements and Disjunct Spatial Use of Harbor Seals (*Phoca vitulina*) in the Inland Waters of the Pacific Northwest

**DOI:** 10.1371/journal.pone.0039046

**Published:** 2012-06-18

**Authors:** Sarah H. Peterson, Monique M. Lance, Steven J. Jeffries, Alejandro Acevedo-Gutiérrez

**Affiliations:** 1 Department of Biology, Western Washington University, Bellingham, Washington, United States of America; 2 Wildlife Science Program, Washington Department of Fish and Wildlife, Lakewood, Washington, United States of America; University of Plymouth, United Kingdom

## Abstract

**Background:**

Worldwide, adult harbor seals (*Phoca vitulina*) typically limit their movements and activity to <50 km from their primary haul-out site. As a result, the ecological impact of harbor seals is viewed as limited to relatively small spatial scales. Harbor seals in the Pacific Northwest are believed to remain <30 km from their primary haul-out site, one of several contributing factors to the current stock designation. However, movement patterns within the region are not well understood because previous studies have used radio-telemetry, which has range limitations. Our objective was to use satellite-telemetry to determine the regional spatial scale of movements.

**Methodology/Principal Findings:**

Satellite tags were deployed on 20 adult seals (n=16 males and 4 females) from two rocky reefs and a mudflat-bay during April–May 2007. Standard filtering algorithms were used to remove outliers, resulting in an average (± SD) of 693 (±377) locations per seal over 110 (±32) days. A particle filter was implemented to interpolate locations temporally and decrease erroneous locations on land. Minimum over-water distances were calculated between filtered locations and each seal's capture site to show movement of seals over time relative to their capture site, and we estimated utilization distributions from kernel density analysis to reflect spatial use. Eight males moved >100 km from their capture site at least once, two of which traveled round trip to and from the Pacific coast, a total distance >400 km. Disjunct spatial use patterns observed provide new insight into general harbor seal behavior.

**Conclusions/Significance:**

Long-distance movements and disjunct spatial use of adult harbor seals have not been reported for the study region and are rare worldwide in such a large proportion of tagged individuals. Thus, the ecological influence of individual seals may reach farther than previously assumed.

## Introduction

Harbor seals (*Phoca vitulina*) are the most abundant breeding pinniped species in the Pacific Northwest [1]. While considered non-migratory [2], harbor seals travel varying distances from a primary haul-out site and the distance moved from a haul-out site provides a measure of the maximum space over which behaviors, including foraging and mating, can occur.

Worldwide, adult harbor seals typically limit their movements and activity to <50 km from their primary haul-out site [1], [3]–[9]. Many of these results come from studies that used very high frequency (VHF) radio-telemetry, which is limited in its ability to track animals over large spatial scales for extended periods of time or monitor animals continuously once they leave a given study area. Consequently, in many regions it is unknown how far seals move when they are out of radio-telemetry range or are not being monitored. Although some VHF tracking studies documented seals moving over larger distances, including an adult seal that moved >220 km (one-way) in Oregon [9] and three seals that moved >200 km (one-way) in central California [10], [11], the proportion of individuals in each of these studies that moved >100 km was small. Moreover, VHF radio-telemetry studies were unable to document continuous movement tracks or the speed at which movements occurred. VHF radio-telemetry studies in the Pacific Northwest did not observe seals >30 km from capture sites [1], [3], [12], [13]; however, there were periods of time when seals could not be located suggesting that tagged seals were outside of the study area. The use of satellite-telemetry in eastern Canada, Alaska, and Scotland allowed scientists to accurately quantify the timeframe for adult harbor seal movements, observing one-way movements up to 520 km [14], 197 km [15] and 144 km [16]. It is then possible that at least some seals move long distances in the Pacific Northwest as well, behavior that could be revealed through the regional use of satellite telemetry. Given the potential impact of harbor seals to commercially-important fish species in the Pacific Northwest [17], [18], it is important to establish the spatial scale at which such impact may occur.

Based on differences in pupping phenology [19] and genetic variation of mitochondrial DNA [20], federal resource managers have divided harbor seals of the U.S. Pacific Northwest into two distinct stocks, one that includes the coastal waters (Oregon and Washington Coastal Waters Stock) and one that includes the inland marine waters (Washington Inland Waters Stock) [21]. The stock designation is further supported by previous VHF-radio telemetry studies in the region [1], [3], [12], [13], which did not observe long-distance movements that could connect the two stocks. The dual stock designation assumes that male and female harbor seals move similarly, as mitochondrial DNA analyses do not detect male-mediated gene flow. In contrast, microsatellite DNA analyses support the hypothesis that male and female harbor seals have different rates of gene flow between populations in the Pacific Northwest, suggesting differences in movements between males and females [22]. Further, high rates of male-mediated gene flow, previously undetected by mitochondrial DNA analyses, were observed between management stocks of Alaska harbor seals [23]. Although the results from genetic studies suggest some movement by males between the regions occupied by the two U.S. Pacific Northwest stocks, to date no such movement has been detected.

**Figure 1:**
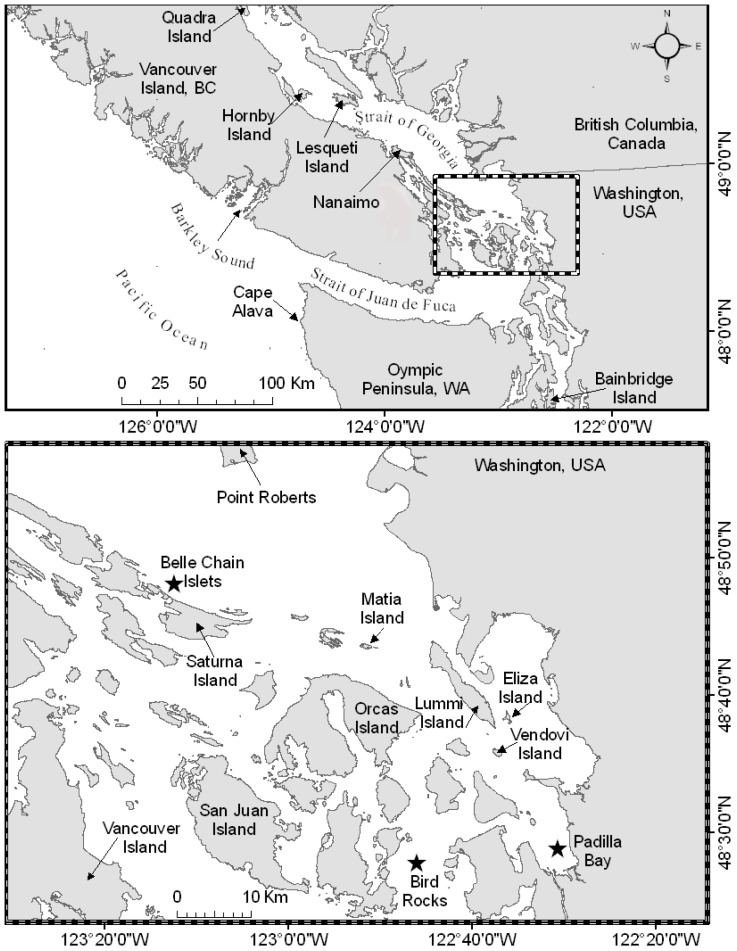
Study site. Top map: the study site within the Pacific Northwest. Inset and lower map: harbor seal capture sites indicated by a star.

Satellite telemetry has been used extensively on pinniped species to continuously record geographic locations without range limitations [14], [24]–[27], and does not noticeably alter animal behavior [28], [29]. Locations obtained from satellite telemetry can be used to examine two-dimensional movement patterns and quantify regions of higher use through kernel density estimates [30]. We used satellite tags to observe adult harbor seal movements within the inland waters of the Pacific Northwest (Fig. 1) to determine the scale of their movements and outline potential implications for spatial use and gene flow. Seals were captured at haul-out sites adjacent to rocky or soft-bottomed habitats, representative of two predominant habitats found within the region. To quantify movements we attached satellite transmitters to 20 adult seals and collected satellite-derived locations between April and October 2007. We documented movement patterns that have not been previously observed in adult harbor seals from the Pacific Northwest and provide new insight into harbor seal behavior.

## Methods

### Ethics statement

The animal use protocols used in this research were reviewed and approved by the Institutional Animal Care and Use Committee at Western Washington University (Protocol Number 06-005) and at the National Marine Mammal Laboratory (for Marine Mammal Protection Act Scientific Research Permit 782–1702). This research was conducted under the Marine Mammal Protection Act Scientific Research Permit 782–1702 issued to the National Marine Mammal Laboratory by NOAA's Protected Resources Division and under a Department of Fisheries and Oceans Research License.

### Captures and satellite tag deployment

Adult harbor seals were captured during April and May 2007 at three sites (Table 1, Fig. 1), at least two months prior to the August peak of parturition in the inland waters of the Pacific Northwest [1]. Capture sites were located in Padilla Bay, with characteristic estuarine-mudflat haul-outs (48°28.37′N, 122°30.88′W), and Bird Rocks, with three clustered intertidal rocky reef haul-outs in Rosario Strait (48°29.16′N, 122°45.61′W), in the eastern San Juan Islands, Washington, USA. The Belle Chain Islets (Belle Chain) are a cluster of intertidal rocky reef haul-outs in the southeastern Gulf Islands of British Columbia, Canada (48°49.67′N, 123°11.56′W). All capture sites are tidally influenced and used regularly throughout the year. We assumed that the sex ratios at each haul-out site were similar and animal captures were random, based on past experience using the following techniques and gear.

**Table 1:** Harbor seals captured in April and May 2007 at three haul-out sites in the Pacific Northwest.

Capture	Seal	Deploy	Mass	Length	Total	Total	Standard	Standard	Maximum	Median	Number of
site	ID	date	(kg)	(days)	prefiltered	filtered	prefiltered	filtered	displacement	displacement	core areas
					locs/day	locs/day	locs/day	locs/day	(km)	(km)	
Bird Rocks	Y1455	4/4/07	76.5	135	10.0	5.3	0.9	0.9	11.2	5.2	1
Bird Rocks	B1695	4/5/07	71.5	156	9.7	7.3	2.4	2.2	129.6	105.9	1
Bird Rocks	B1696	4/4/07	74.5	58	10.9	8.8	2.7	2.5	280.9	69.8	2
Bird Rocks	B1697	4/6/07	96.0	94	5.3	3.5	0.5	0.5	186.2	97.2	5
Bird Rocks	B1698	4/6/07	90.0	83	5.2	3.0	0.6	0.5	44.0	1.8	1
Bird Rocks	B1701	4/20/07	86.0	179	11.7	8.6	2.3	2.2	139.9	19.0	1
Padilla Bay	Y1459	4/19/07	83.0	134	8.7	5.8	2.0	1.8	41.6	15.4	1
Padilla Bay	Y1460	4/19/07	62.5	101	2.4	1.7	0.4	0.4	32.8	3.5	1
Padilla Bay	Y1462	5/21/07	77.5	116	8.6	5.3	1.2	1.1	6.0	1.8	1
Padilla Bay	B1699	4/18/07	64.0	147	11.5	9.9	4.8	4.7	18.6	2.6	1
Padilla Bay	B1712	5/21/07	69.0	107	9.2	7.6	4.3	4.1	9.6	5.6	1
Padilla Bay	B1713	5/21/07	54.0	113	10.2	8.2	3.1	2.9	116.6	2.2	1
Belle Chain	B1702	5/1/07	81.5	76	6.1	3.9	2.0	1.9	23.3	1.8	1
Belle Chain	B1703	5/1/07	66.5	126	12.7	7.0	1.6	1.4	49.2	13.5	2
Belle Chain	B1704	5/1/07	72.0	97	6.9	4.6	1.9	1.8	218.0	11.7	2
Belle Chain	B1706	5/1/07	90.5	132	9.0	6.7	2.1	2.0	16.6	5.0	2
Belle Chain	B1707	5/2/07	58.5	102	7.3	5.6	2.0	1.9	216.6	17.7	3
Belle Chain	B1709	5/3/07	92.0	97	8.6	6.0	1.3	1.1	33.8	12.5	1
Belle Chain	B1710	5/3/07	77.0	46	8.9	6.0	2.3	2.1	35.8	6.0	1
Belle Chain	B1711	5/3/07	70.5	99	9.4	6.9	2.0	1.8	137.8	15.0	2

Note. Seal ID indicates female (Y) or male (B) followed by a number unique to that individual. Length represents the total number of days from tag deployment to tag failure for each animal. Standard locs per day is the mean number of filtered 1, 2 and 3 quality level locations per day for each seal. Maximum displacement is the greatest over-water distance traveled by each seal from the capture site and mean distance is the average distance between all standard locations for each seal and the capture site. Number of core areas is the number of distinct regions identified by the 50^th^ percentile contours from kernel density estimates.

Seals were captured using several methods, including boat rushes, beach seines and tangle-nets [31]. Captures in Washington were led by the Washington Department of Fish and Wildlife (WDFW) and in Canada by the Department of Fisheries and Oceans (DFO). After entanglement in a net, using standard protocols [31], seals were physically restrained while being sexed, weighed, and measured (standard length). Seals that weighed >50 kg were classified as adults, based on regional research by Bigg [32], and selected for electronic tag instrumentation. In addition, all animals were tagged on each hind flipper with a uniquely numbered Dalton tag for future identification.

Satellite tags were deployed on six harbor seals from each of the Washington sites and eight seals from Belle Chain (Table 1). SPOT5 satellite tags (Wildlife Computers, Redmond, Washington, USA) were glued to the heads of all seals from Padilla Bay and Bird Rocks, and SPLASH tags (Wildlife Computers) were glued to the upper backs of all seals from the Belle Chain Islets, in all cases using five-minute epoxy [31]. SPOT5 satellite tags were solely platform transmitter terminals (PTTs), which only allowed calculation of location, while SPLASH tags included both PTTs and time-depth recorders. Placement of SPOT5 and SPLASH tags differed due to their sizes; SPLASH tags required a larger area on the seal due to the need to place them in a retrievable float pack in order to recover time-depth data. SPOT5 tags were programmed to pause transmissions after the tag was dry (haul-out state) for one hour and SPLASH tags were programmed to pause transmissions after the tag was dry for two hours. Transmissions resumed when the tag was wet for >20 s (SPOT5) or >30 s (SPLASH) within a minute. Additionally, SPOT5 tags were programmed for an alternating duty cycle of two hours on and one hour off, and SPLASH tags were programmed for an alternating duty cycle of four hours on and one hour off. Satellite tags transmitted until they were shed during the annual molt or until the tag either malfunctioned or ran out of battery power. The mean number of filtered standard, auxiliary and total locations per day did not differ significantly between SPOT5 and SPLASH tags (Kruskal-Wallis, χ^2^=0.073, p=0.787; ANOVA, F=0.082, p=0.778; and Kruskal-Wallis, χ^2^=0.292, p=0.589; respectively). Hence, we analyzed locations obtained by both tag types in the same manner and compared the results.

### Data analysis

Satellite-derived locations were obtained using the Argos data collection system, which assigns a location quality based on the number of uplinks received by a passing satellite [33], although the error may be greater than that reported by Argos [34]. Satellite-derived locations were processed using several steps to remove erroneous locations and interpolate movement tracks to obtain locations at equal time intervals. First, secondary Argos locations were examined manually and swapped if the secondary location was closer to the previous and subsequent locations than the primary location [35]. Locations were run through a speed-distance-angle filter using the sdafilter function in the R package “argosfilter” [36] with a 2 m/sec swim speed threshold [4], [37], [38] and the default parameters for turn angle (15, 25) and distance (2500, 5000) to remove improbable auxiliary and standard locations. A particle filter was applied to the remaining locations, which both interpolated locations to time intervals of every 240 minutes (6 locations per day) and significantly decreased the number of locations falling on land for the majority of seals [39]. Refer to the supporting material for a more detailed description (Text S1, Fig. S1). Mean (± SD) errors for 0, A and B quality Argos locations estimated from a study with paired Argos and Fastloc GPS tags deployed on *Zalophus californianus* were 3.87 (±5.59), 4.41 (±6.47), and 7.67 (±10.80) km, respectively [34]. Mean (± SD) errors for 1, 2, and 3 quality Argos locations were estimated to be 1.05 (±1.01), 0.95 (±1.00), and 0.60 (±0.56) km, respectively [34]. These estimates of Argos error were used as the error structure for the particle filter.

Filtered tracks were input into ArcView 10 (Environmental Systems Research Institute Inc., Redlands, California, USA), which was used to measure over-water distances traveled by each seal and calculate kernel density estimates. The minimum over-water distance between each location and the site where a seal was captured was calculated to obtain sequential distances between satellite locations and the capture site. Over-water distances were obtained using the Cost Distance tool in ArcView 10 (Spatial Analyst tools) where land was made so costly that the least costly path between locations was kept over water and forced around land masses. Refer to the supporting material for more detail (Text S2). We calculated maximum and median over-water distances between the capture site and all locations for each seal. Fixed kernel density estimates were performed using the Kernel Density tool (Spatial Analyst tools) to generate a probability density estimate, interpreted as a utilization distribution [40]. A bandwidth of 2500 m (h) was chosen for the study population to capture movements of all seals without under-smoothing animals that moved over greater distances. The appropriateness of this bandwidth was determined by conducting the analysis with multiple bandwidths (Kernel Density tool) and visually inspecting the results for similarity [40], [41]. Bandwidth selection was not critical for this study since we did not quantify absolute size of home ranges or core areas but were instead interested in large-scale differences in spatial use patterns and the use of disjunct core areas. We established 75^th^, 50^th^ and 25^th^ percentile contours from the utilization distribution to examine the number of distinct regions used by each seal and the 50^th^ percentile contours were specifically used to quantify the number of core areas for each seal [30].

## Results

### Satellite transmissions

Satellite tags transmitted a mean (± SD) of 110 (±32) days with a range of 46–179 days (Table 1). After speed-distance-angle filtering, individual seals had a mean 693 (±377) total locations, 214 (±160) of which were standard quality locations. Standard quality locations accounted for an average 29.6 (±11.3)% of filtered locations. After the particle filter was applied, seals had a mean of 654 (±194) total locations.

### Movements

Overall, 14 of 20 seals, 12 of which were males, moved farther from their capture haul-out site than previously observed in the region (Table 1). Females moved relatively shorter distances than most males; the maximum distance moved by females in this study was significantly less than males (Kruskal-Wallis, χ^2^=3.938, p=0.047). Maximum over-water distances from the capture site for male harbor seals ranged 9.6–280.9 km (mean=103.5, SD=87.0) while females ranged 6.0–41.6 km (mean=22.9, SD=17.0) (Table 1). Median over-water distances between satellite locations and the capture site for all seals ranged 1.8–105.9 km (mean=20.7, SD=31.4) (Table 1). Males had median distances from their capture site ranging 1.8–105.9 km (mean=24.2, SD=34.3), while females had median distances ranging 18–15.5 km (mean=6.5, SD=6.1) (Table 1). Median distances from the capture site were not significantly different between males and females (Kruskal-Wallis, χ^2^=0.893, p=0.345).

**Figure 2:**
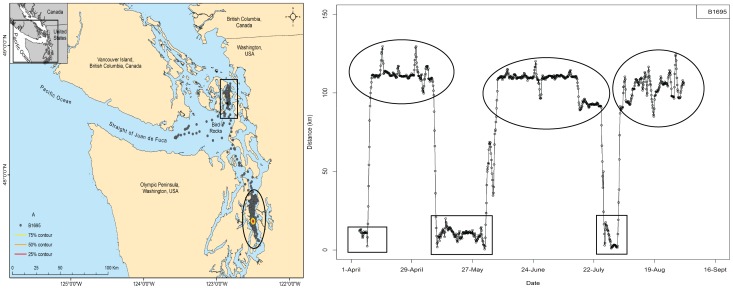
Reference panel of paired map and minimum over-water distance traveled. Reference panel showing a map of locations for one seal (left side) paired with the corresponding minimum over-water distances between sequential satellite locations and the capture site over the course of the study (right side). The map has an inset of the entire study area. Over-water distance figures are labeled every four weeks on the x-axis. Rectangles indicate locations around Bird Rocks and ellipses indicate locations around south Puget Sound (Bainbridge Island). 75^th^, 50^th^ and 25^th^ percentile contours from the utilization distribution (generated by kernel density analysis) are shown on the left.

**Figure 3:**
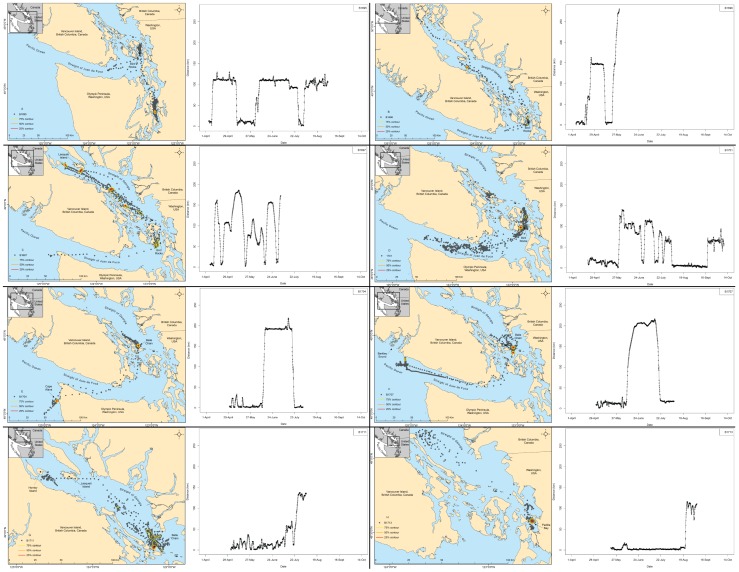
Paired maps and minimum over-water distances traveled for seals that moved >100 km. Paired maps of satellite locations, contours, and movement plots of the minimum over-water distance between sequential satellite locations and the capture site for seals that moved >100 km away from their capture site over the course of the study. All seals were males from Bird Rocks or Belle Chain. Each seal has one panel and panels are labeled in the legend of each map.

**Figure 4:**
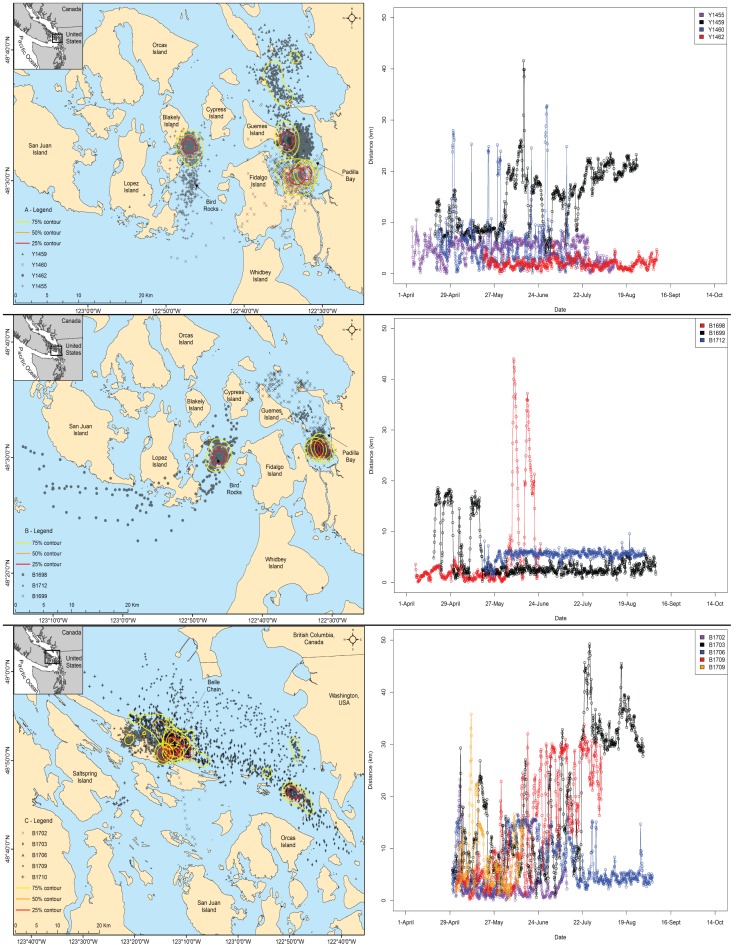
Paired maps and minimum over-water distances traveled for seals that moved <100 km. Paired maps of satellite locations, contours and movement plots of the minimum over-water distance between sequential satellite locations and the capture site for seals that moved <100 km away from their capture site over the course of the study. Multiple seals are represented on each map and over-water distance figure. Each seal has a different symbol on the maps and different colors on the over-water distance figures. Female seals are represented in panel A, male seals captured at Padilla Bay and Bird Rocks are represented in panel B and male seals captured at Belle Chain are represented in panel C.

Movement and spatial use within the study region by individual seals can be visualized in Figures 2, 3 and 4. Refer to Figure 2 as a reference for interpreting these paired figures. Figure 2 shows an individual that moved between two regions >100 km apart. Each dot on both the map and distance plot is one interpolated location. Figure 2 shows that this seal, B1695 from Bird Rocks, primarily used two disjunct regions and moved rapidly between them, at times traveling >100 km in just over 2 days. Circles and squares highlight the two separate geographic regions used by this seal, the capture region enclosed in a square. Colored contour lines indicate the 75^th^, 50^th^, and 25^th^ percentile contours from the utilization distribution generated by the kernel density estimates and indicate that this seal had one core area (50^th^ percentile contour).

One-way distances >100 km were observed for eight of the 16 males (Table 1, Fig. 3). Harbor seals traveling distances >100 km from their haul-out site exhibited rapid movements between start and end locations, with individual males covering upwards of 100 km in as little as two days (Fig. 3). Male harbor seals made movements that spanned the majority of the inland waters of the Pacific Northwest ranging west to the Pacific Ocean, to the northern reaches of the Strait of Georgia and into southern Puget Sound; however, none of the seals moving over large distances moved to the same places, indicating variability in spatial use of the region. Spatial segregation of movements was demonstrated by seals from Bird Rocks that moved into different bodies of water. One male seal used primarily the Strait of Georgia (north) as a secondary location, while another male used southern Puget Sound (south) as a secondary location, and a third male used the Strait of Juan de Fuca (west) as a secondary location. Locations and islands mentioned in regards to movements can be viewed in Figure 1.

Generally, seals that moved >100 km remained in the new location for 1–8 weeks and six of these seals returned at least once to their capture site (Fig. 3). One male from Bird Rocks (B1695) traveled >100 km south from Bird Rocks to southern Puget Sound on three separate occasions (Fig. 3A) while another male from Bird Rocks (B1696) traveled north into the islands west of Belle Chain on one trip and then to Quadra Island at the northern end of the Strait of Georgia, Canada (Fig. 3B). B1697 traveled to Lesqueti and Hornby Islands in British Columbia, Canada, as well as to the outer coast of Washington State, USA (Fig. 3C). B1701 traveled to Belle Chain, the Strait of Juan de Fuca and throughout the San Juan Islands (Fig. 3D). B1697 and B1701, captured at Bird Rocks, both made five lengthy trips. Two seals from Belle Chain (B1704 and B1707) also traveled to the outer coast of Washington and British Columbia, >200 km each way, and remained on the coast for over a month (Fig. 3F). The only seal from Padilla Bay that made lengthy movements was a male (B1713) that traveled north of Belle Chain at the end of August and remained there until his tag fell off in early September (Fig. 3H). Several individuals, such as the two males from Belle Chain, demonstrated similar trips to each other, in both timing and distance. However, visual comparison of movement figures for all seals did not reveal any overt patterns in the locations where harbor seals moved away from their capture site, the distance they traveled away from their capture sites or the timing when these movements were undertaken.

### Concentrated areas of use

All 20 seals used the space adjacent to their capture haul-out site for varying lengths of time. While we cannot be entirely sure that the capture site was the primary haul-out site for a captured animal, all but one seal in the present study had a portion of their core area (50^th^ percentile contours) within 10 km of the capture site. Individual seals had between one and five core areas (50^th^ percentile contours from utilization distributions generated from kernel density estimates) (Table 1). Disjunct regions (>100 km apart) were used by eight males from two rocky reef sites, four of which also had disjunct core areas (Fig. 3). Disjunct core areas (>100 km apart) were demonstrated for two males from Bird Rocks (B1696 and B1697) and two males from Belle Chain (B1704 and B1707), and there was no overlap for these animals in core areas used away from the capture sites (Fig. 3). Individual seals moving <100 km used unique locations away from their capture site; however there was much greater overlap in their 75^th^, 50^th^ and 25^th^ percentile contours than in seals that traveled >100 km (Fig. 4). Seals captured in Padilla Bay all had their core areas in the vicinity of their capture sites, within the confines of Padilla Bay. Only one seal, female Y1459, had a portion of her 75^th^ percentile contour outside of Padilla Bay (Fig. 4A). There was a high degree of overlap for locations and core areas for these six seals unlike seals from Bird Rocks or Belle Chain (Fig. 4).

## Discussion

Our study documented important and previously unreported elements of regional harbor seal behavior that seldom have been reported worldwide as well. Individual harbor seal movements covered a larger area than previously thought and some individuals had multiple activity centers, indicating that seals are using space in a more complex manner than previously assumed. In the present study, 14 satellite tagged harbor seals had a maximum over-water distance from their haul-out site greater than the maximum distance previously observed in the region [3], [12], [13]. Of these 14 seals, eight seals moved distances >100 km and kernel density estimates identified core areas use (50^th^ percentile contours) separated by >100 km for four seals. We believe that long-distance movements of that magnitude strongly suggest consumption of prey in these disjunct regions, as suggested by other studies [9], [14], [15], [42]; therefore the foraging impact of an individual seal may occur over a wider geographic scale than previously assumed. Our results also support the hypothesis based on genetic studies [22] that male harbor seals move between the two regional stocks: Oregon and Washington Coastal Waters Stock and Washington Inland Waters Stock. Because parturition overlaps between the Coastal and Inland stocks [1], male harbor seals that moved to the outer coast could potentially mate in multiple locations and provide some gene flow between the two seal stocks.

We tagged adult harbor seals during late spring and summer; therefore it was surprising to see repetitive movements >100 km for such a large proportion of animals. In other regions, harbor seals traveled distances >100 km; however, such movements are more commonly observed for juveniles [9], [15], indicative of seasonal movements to over-wintering sites [14], or observed in a small percentage of the total sample size [10], [11], [43]. Males in the Saint Lawrence River Estuary moved up to 520 km between summer and wintering sites but were limited in their movements during the middle of a season: 90% of standard satellite locations were <10 km from their summer haul-out sites [14]. The longest trip duration other than the seasonal switch in haul-out sites was 12 days [14]. We found six adult males had round-trip movements >200 km that lasted 7–56 days between April and August (Fig. 3) and ended within 10 km of the capture site, indicating fidelity to the capture region. Our research suggests that adult harbor seal movements are more complex than previously described and that within the Pacific Northwest seals can move large distances and use disjunct locations. We were unable to fully examine the influence of sex on movements due to the small and unequal number of females in the present study; therefore this question should be addressed by future research.

Multiple harbor seals demonstrated disjunct regions of use, suggesting individual spatial preference for certain areas within the region over others. Preferential use of certain habitats or a response to spatio-temporal changes in prey density may partially explain the movement patterns observed in the present study. Previous research observed differences in harbor seal movements, as well as diving and foraging behavior, relative to the different habitats and habitat-specific prey availability adjacent to their capture site [7], [44], [45]. Prey resources and harbor seal diet differ between habitats in the Pacific Northwest [46], [47], and harbor seals may have moved deliberately to exploit reliable, yet ephemeral regions of higher prey abundance. Movements (<50 km) for seals along the coast of Oregon and Washington have been attributed to seasonal fluctuations in prey abundance [9], , and movements of 125 km by adult females in Alaska coincided with eulachon runs in the Copper River Delta [15]. Harbor seals from rocky reef sites in northern Puget Sound prey primarily upon Pacific herring (*Clupea pallasi*) and adult salmonids [46], [48], both of which demonstrate significant seasonal regional shifts in abundance and distribution [49], [50]. Changes in the distribution of these prey species in the Pacific Northwest may influence harbor seal foraging behavior [51] and spatial distribution [18] and help explain the movements that we observed. Harbor seals aggregate around salmonid prey pulses [17], [52]–[54] and seals in the present study may have been utilizing locations within the inland waters where prey species such as salmonids aggregate ephemerally. Regions used on the outer coast likely corresponded with annual regions of increased productivity at the convergence of the Pacific Ocean and the Strait of Juan de Fuca [55], [56]. Based on the speed at which a seal moved between locations, movements >50 km by satellite tagged seals in the present study appeared to be directed movements and not random walks [57], suggesting that the seals moved deliberately between locations.

In contrast to seals traveling >100 km to disjunct regions, all seals from Padilla Bay (n=3 males, 3 females) had only one core area adjacent to their capture site. Diet analysis of harbor seal scats from Padilla Bay [47] and adjacent haul-out sites [46], [48] revealed the presence of a wide diversity of smaller estuarine prey items, suggesting that seals from Padilla Bay mostly foraged within the estuary on continuously abundant estuarine prey species. Characteristics that make Padilla Bay a prominent nursery area [12], [58], such as the presence of benthic prey important to recently weaned pups, including sand shrimp, sculpins and flatfish, and other habitat characteristics may contribute to the localized movements observed. The habitat adjacent to rocky reef and estuarine mudflat-bay haul-out sites may be a contributing factor dictating movements and spatial use of harbor seals in the region; therefore we suggest that future studies should examine a possible influence of haul-out habitat characteristics at both tagging and destination sites.

Inland and coastal harbor seals have been separated into multiple distinct stocks based on differences in both the timing of pupping and mitochondrial DNA, which is maternally inherited and suggests that there is limited movement by female harbor seals [1], [20], [59]. Based on the lack of exchange of radio tagged seals between these two stocks, it was believed that the stocks do not mix [21] and therefore movement between the inland waters and the outer coast was considered unlikely. However, microsatellite analysis shows less separation between inland and coastal populations than mitochondrial analysis [22], indicating that males may be an undetected mechanism for gene flow to occur between these groups. Male-mediated gene flow between harbor seal stocks was recently documented in Alaska [23] and satellite-telemetry in Scotland documented seals moving between separately defined populations [16]. Our results provide a potential mechanism for low levels of paternal gene flow between stocks within the Pacific Northwest. Although we cannot be sure of the reasons behind movements to the outer coast, the timing of movements places several males on the outer coast during and after peak parturition [1], when mating likely occurs [60]. Seals that travel to the outer coast for mating and then return to the inland waters would potentially be able to mate with females from multiple stocks.

An alternate hypothesis to explain the short- and long-term movements that we detected is response to vessel disturbance. However, it is unlikely that the rapid and directed movements in the present study can be explained by disturbance from recreational vessel traffic because previous research at a rocky reef site in the region observed that the majority of disturbance events resulted in full recovery to pre-disturbance levels within three hours [61].

The results and novel observations from our research, specifically that harbor seals moved greater distances than previously documented and demonstrated use of disjunct regions, add to our understanding of harbor seal behavior within the region and worldwide. While we are unable to link observed movements to a specific cause, we speculate that these movements may be driven by prey distribution and foraging opportunities as well as the potential for mating opportunities. Some harbor seals, considered central place foragers, could have multiple disjunct locations in the Pacific Northwest from which they base foraging trips. Additionally, males traveling between the outer coast and inland waters at the appropriate times could take advantage of the staggered timing of mating and mate in both regions. Therefore, based on our observations, the ecological impact of individual harbor seals may be spread over a much wider area in the Pacific Northwest than previously assumed.

## Supporting Information

Figure S1 **Filtering method visually represented for one seal.** Locations in dark gray were removed by the speed-distance-angle filter and locations in blue were removed by the particle filter, leaving the locations in yellow to be analyzed.
(TIF)

Text S1 **This supplemental text is a description of the particle filter that was applied to satellite-derived locations as part of the pre-analysis filtering protocols.**
(DOC)

Text S2 **This supplemental text is a description of the process in ArcView 10 that was used to obtain over-water distances.**
(DOC)
