# Supplementary figures and images for: Long Distance Movements and Disjunct Spatial Use of Harbor Seals (Phoca vitulina) in the Inland Waters of the Pacific Northwest
Source: PLoS One. 2012 Jun 18;7(6):e39046. doi: 10.1371/journal.pone.0039046 (PMC3377613; doi:10.1371/journal.pone.0039046)

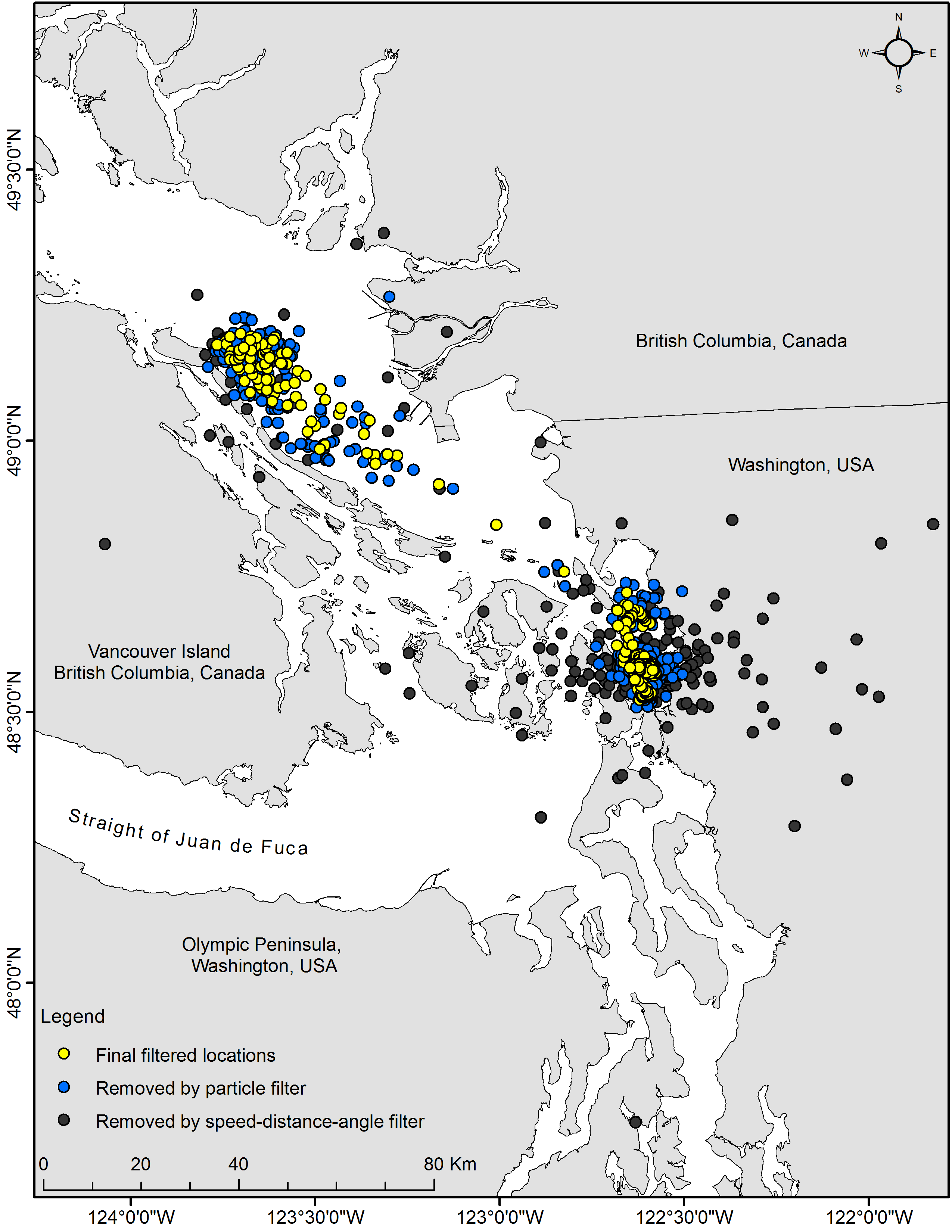

Supplement: Figure S1 — Filtering method visually represented for one seal. Locations in dark gray were removed by the speed-distance-angle filter and locations in blue were removed by the particle filter, leaving the locations in yellow to be analyzed. (TIF) [file pone.0039046.s001.tif]
