# Supplementary material for: Long Distance Movements and Disjunct Spatial Use of Harbor Seals (Phoca vitulina) in the Inland Waters of the Pacific Northwest
Source: PLoS One. 2012 Jun 18;7(6):e39046. doi: 10.1371/journal.pone.0039046 (PMC3377613; doi:10.1371/journal.pone.0039046)
Supplement: Text S1 — This supplemental text is a description of the particle filter that was applied to satellite-derived locations as part of the pre-analysis filtering protocols. (DOC) [file pone.0039046.s002.doc]

### Supporting Information – S1

### Data analysis

**Pre-filtering steps.** The Argos system provides two possible locations for certain transmissions and the second latitude and longitude pairs were examined to see if they provided a better solution than the primary Argos provided solution. The coordinates were switched if it was obvious that the primary Argos solution was biologically improbable but the second Argos solution was biologically reasonable. Additionally, the lower quality of two transmissions occurring ≤ 60 s of each other was removed, and if both transmissions were of equal quality then the second transmission was removed. At this point, filtering methods were implemented to remove likely erroneous points from the data set.

Filtering methods**.** Locations were first filtered using the argosfilter package in R . The maximum speed threshold was set at 2m/s and the default parameters for turn angle (15, 25) and distance (2500, 5000) were utilized. The filtered locations were then run through a particle filter written , which interpolated locations to one every 240 min and reduced the number of locations on land for most animals (Fig. S1). Twenty-five particles were generated for every filtered satellite location, however particles were not generated over space designated as land using the full resolution GSHHS (global self-consistent, hierarchical, high-resolution shoreline database) coastline . The particle filter was iterated 25 times, which is more than the minimum number of suggested iterations . The particle filter resulted in a decrease in locations on land for the majority of cases when animals were transiting between land masses in the study region. One notable exception was in the case of seal Y1460, when more locations were put onto the adjacent land mass. This seal was fairly resident and had the lowest number of locations/day, which resulted in the particle filter assigning more locations over the peninsula of land adjacent to the bay, which may not have been identified properly as land using GSHHS. However, this assignment of location did not impact the results or conclusions drawn from this study and this method was determined to be a robust method to account for Argos error and obtain locations at equal time intervals.

**References**

1. Freitas C, Lydersen C, Fedak MA, Kovacs KM (2008) A simple new algorithm to filter marine mammal Argos locations. Marine Mammal Science 24: 315-325.

2. Williams TM, Kooyman GL (1985) Swimming performance and hydrodynamic characteristics of harbor seals *Phoca vitulina*. Physiological Zoology 58: 576-589.

3. Thompson PM, Miller D (1990) Summer foraging activity and movements of radio-tagged common seals (*Phoca vitulina* L) in the Moray Firth, Scotland. Journal of Applied Ecology 27: 492-501.

4. Lesage V, Hammill MO, Kovacs KM (1999) Functional classification of harbor seal (Phoca vitulina) dives using depth profiles, swimming velocity, and an index of foraging success. Canadian Journal of Zoology-Revue Canadienne De Zoologie 77: 74-87.

5. Tremblay Y, Robinson PW, Costa DP (2009) A parsimonious approach to modeling animal movement data. PLoS ONE 4: e4711.

6. Wessel P, Smith WHF (1996) A global, self-consistent, hierarchical, high-resolution shoreline database. J Geophys Res 101: 8741-8743.
