# Supplementary material for: Long Distance Movements and Disjunct Spatial Use of Harbor Seals (Phoca vitulina) in the Inland Waters of the Pacific Northwest
Source: PLoS One. 2012 Jun 18;7(6):e39046. doi: 10.1371/journal.pone.0039046 (PMC3377613; doi:10.1371/journal.pone.0039046)
Supplement: Text S2 — This supplemental text is a description of the process in ArcView 10 that was used to obtain over-water distances. (DOC) [file pone.0039046.s003.doc]

### Supporting Information – S2

### Data analysis

**Over-water distance analysis.** Over-water distance calculations were done using the Cost Distance tool in the Spatial Analyst Toolbox. The study area was first converted to a raster with 100 m grid cells and reclassified so that each grid cell of water received a value of 1 and each grid cell of land received a value of 1,000,000. Four separate cost-distance analyses were conducted to obtain cost-distance values for every grid cell from the four capture sites (Belle Chain, Bird Rocks, and two sites in Padilla Bay). Quantifying land with such a high value keeps paths over water for as long as possible. The path remains over water until it reaches the closest point where land and water meet and then runs the path over land to each land grid cell. The Extract to Points tool was used to obtain cost-distance values for every location of each seal, using the appropriate cost-distance raster based on their capture site. Cost-distance values for locations over water were equivalent to kilometer distances; however the cost-distance values for locations on land needed further conversion to be interpreted as actual distances. Cost-distance analysis was conducted again, using a value of 0 for water instead of 1, which resulted in the cost-distance involved in moving over land to land grid cells. Over-land cost-distance values were subtracted from the entire cost-distance value for grid cells on land, to obtain separate cost-distance values for the land and water portions of the path. Cost-distance values for grid cells falling on land were divided by 1,000,000 to obtain the actual distance of that path over land in kilometers and the land and water distances were summed to obtain the full path length for that grid cell falling on land.
